# Bibliography

**Published:** 1899-03

**Authors:** 


### Bibliography.

Annual and Analytical Cyclopædia of Practical Medicine. By Charles E. de M. Sajous, M.D., and One Hundred Associate Editors, assisted by Corresponding Editors, Collaborators, and Correspondents. Illustrated with Chromo-Lithographs, Engravings, and Maps. Vol. II. The F. A. Davis Company, Publishers, Philadelphia, 1899.

The first volume of this most valuable publication was reviewed in a previous issue. This, the second volume, covering subjects from "Bromide of Ethyl" to "Diphtheria," fully confirms the views then expressed. The work as it stands is probably without a rival in medical literature, and the two volumes now placed before medical readers indicate throughout that when the series is completed it will be the most valuable work of reference on medical subjects attempted in this generation.

The effort of the able editor, Dr. Sajous, has been to bring every subject considered fully up to the present period, and for this purpose he has availed himself of the aid of the best authorities on special subjects. In illustration of this care, the following quotation from the preface of the editor will give an idea of the scope of the work and its value.

"The readers will have before them in this volume exceptionally valuable articles on a number of exacting subjects,—namely, 'Cerebral Hemorrhage,' by Dr. William Browning, of Brooklyn; 'Cirrhosis of the Liver,' by Professor Adami, of Montreal; 'Cholera,' by Professor Rubino, of Naples; 'Cholelithiasis,' by Professor Graham, of Toronto; 'Diabetes,' by Professor Lepine, of Lyons, etc. The better-known affections have also been edited by writers of special ability. Among the articles of this kind is that on 'Diphtheria,' by Drs. Northrup and Bovaird, of New York, who contribute a masterly review of our present knowledge of this affection from every stand-point; the papers of Professor Eskridge, of Denver, on 'Catalepsy;' Professor Bondurant, of Mobile, on 'Chorea;' Dr. Norman Kerr, of London, on 'Cocainomania;' Dr. Oliver, of Philadelphia, on 'Cataract;' Professor Nathan S. Davis, of Chicago, on 'Constipation;' Dr. Vickery, of Boston, on 'Dilatation of the Heart.'"

From the article of Dr. Norman Kerr, of London, the following quotation is made upon the treatment of "Acute Cocaine-Poisoning." Dentists are using this agent to a very great extent for various purposes, and oftentimes with a very limited conception of the risks taken, and, in the majority of cases, would be at a loss as to treatment. For such this instruction of Dr. Kerr should have a special value:

"If the poison has been swallowed, the stomach syphon-tube should be at once applied and the contents of the organ evacuated. The patient should be placed in a horizontal position on his back. Tannic acid, iodine, or charcoal may be given as possible chemical antidotes. Stallard advises the stimulation of respiration and circulation by flicking the chest and face with hot and cold towels, as in opium-poisoning; but I cannot say that I have seen benefit from this practice unless it has been done lightly and occasionally for a minute or two. Ammonia or ether inhaled, drunk by the mouth, introduced into the rectum, or administered hypodermically, is useful, as also is the administration of caffeine or coffee. The addition of small quantities of alcohol, in the form of five- to ten-drop doses of tincturæ cardamom. comp., spirit of chloroform, or tincturæ lavandulæ comp. (separate or combined), is sometimes serviceable when coffee cannot be easily taken. Chloroform may be inhaled to relieve the spasm. Strychnine, in minute doses (one-one-hundredth of a grain), with or without a couple of drops or so of tincture of digitalis, is also of value. . . .

"When the blood-pressure has been raised, or there is alarming respiratory spasm, a drop dose of nitro-glycerin of intervals of half an hour, if required, sometimes acts excellently. Clifford Allbutt says that the inhalation of oxygen and artificial respiration against the asphyxia may be indicated. I have found sips of hot water, and where this could not be taken by the mouth, on account of insensibility or collapse, hot water enemata, of three to four ounces, of substantial aid. External applications as hot as can be borne, such as bottle or jar, or tin filled with hot water and covered with flannel to protect the skin, I make it a rule always to apply, especially in unconsciousness, and, indeed, almost from the first."

Space will not permit allusion to the valuable articles in detail. The chromo-lithographs and other engravings admirably illustrate the text and add much to the beauty of the volume.

Dentists who feel that they cannot afford a library of medical works in addition to the many now published in their specialty, cannot do better than to add this Cyclopædia to their list of books. It will give them all they need in the way of reference, and will do for them, in a medical direction, what the Century Dictionary accomplishes for the literary or professional writer.

A Dictionary of Dental Science and such Words and Phrases of the Collateral Sciences as pertain to the Art and Practice of Dentistry. By Chapin A. Harris, M.D., D.D.S. Sixth Edition, carefully revised and enlarged by Ferdinand J. S. Gorgas, M.D., D.D.S. P. Blakiston's Son & Co., Philadelphia, 1898.

To review a dictionary, if not the most difficult of tasks, is at least one in which there can be less agreement with the author and more room for criticism than in any other form of literature. Very few, it is imagined, when they refer to a dictionary, think of the labor spent upon every single word with its definition, the latter requiring much thought and careful arrangement to avoid confusing the meaning. This book is no exception, and that the compilation of words has been fairly well done is attested by the fact that the book has sold through five editions and has now reached the sixth.

Dental students require something more than a medical dictionary. They need and must have the definition of technical terms, not to be found in these to any extent. It is, therefore, not surprising that undergraduates, as well as dental practitioners, have turned to the Harris-Gorgas dictionary as a book best adapted to their needs.

While this is true, the dictionary is by no means faultless. The present editor, doubtless, has been handicapped in this and other works of Dr. Harris by the legacy left of much irrelevant matter that has become antiquated. This is apparent in many of the definitions which would be improved by a dress more in harmony with modern ideas.

This dependence upon the past is peculiarly noticeable in the definition of "Gums, Inflammation, Turgescence, Ulceration, and Recession of." Here a very lengthy dissertation is given upon what is, at present, known as Pyorrhœa Alveolaris, and the authority quoted is Koecker, who flourished in the earlier part of the present century. When the author comes to define Pyorrhœa Alveolaris, he refers to "Alveolar Pyorrhœa," and gives the reader but a paragraph upon the subject, and this does not describe the etiology or therapeutics in an understandable manner. The author may be justified in transposing the words comprising this name, but inasmuch as it is recognized in the ordinary form by all dental writers, and has been accepted by the Century Dictionary, it seems a waste of time for the reader, when hunting for Pyorrhœa, to be referred to another page of the volume.

The length of some of the definitions is very objectionable, many extending to several pages. This is an old evil and not confined, by any means, to this dictionary, but it is nevertheless an annoyance, when looking for a definition, to be met with a mass of verbiage that does not clearly define the subject.

While slips in proof-reading are not a legitimate subject for criticism, in a dictionary they may become a source of error in others. Reference is made here to the spelling of the word "bur." Most proof-readers are troubled when they first strike this word, as used in dentistry. Under the head of "Bur-Drills" it is spelt with one "r" while under "Drill-Burrs" it is spelt with two.

While there is much in this book not altogether satisfactory to the critical mind, there is also much that is good and which cannot readily be obtained elsewhere. It has, therefore, a value for students possessed by no other book, and can be cordially recommended as a work well adapted for this class, and doubtless will hold a place here that cannot be filled by any medical dictionary.

The general make-up of the volume is in the usual excellent character of all the publications of the house of Blakiston's Son & Co.

